# Towards (better) fluvial meta-ecosystem ecology: a research perspective

**DOI:** 10.1038/s44185-023-00036-0

**Published:** 2024-02-07

**Authors:** Lauren Talluto, Rubén del Campo, Edurne Estévez, Florian Altermatt, Thibault Datry, Gabriel Singer

**Affiliations:** 1 Department of Ecology, University of Innsbruck, Technikerstrasse 25, 6020 Innsbruck, Austria; 2 Department of Aquatic Ecology, Eawag: Swiss Federal Institute of Aquatic Science and Technology, Überlandstrasse 133, 8600 Dübendorf, Switzerland; 3 Department of Evolutionary Biology and Environmental Studies, University of Zürich, Winterthurerstrasse 190, 8057 Zürich, Switzerland; 4 National Research Institute for Agriculture, Food and Environment (INRAE), 5 Rue de la Doua, 69100 Villeurbanne, France

**Keywords:** Carbon cycle, Limnology, Community ecology, Ecosystem ecology

## Abstract

Rivers are an important component of the global carbon cycle and contribute to atmospheric carbon exchange disproportionately to their total surface area. Largely, this is because rivers efficiently mobilize, transport and metabolize terrigenous organic matter (OM). Notably, our knowledge about the magnitude of globally relevant carbon fluxes strongly contrasts with our lack of understanding of the underlying processes that transform OM. Ultimately, OM processing en route to the oceans results from a diverse assemblage of consumers interacting with an equally diverse pool of resources in a spatially complex network of heterogeneous riverine habitats. To understand this interaction between consumers and OM, we must therefore account for spatial configuration, connectivity, and landscape context at scales ranging from local ecosystems to entire networks. Building such a spatially explicit framework of fluvial OM processing across scales may also help us to better predict poorly understood anthropogenic impacts on fluvial carbon cycling, for instance human-induced fragmentation and changes to flow regimes, including intermittence. Moreover, this framework must also account for the current unprecedented human-driven loss of biodiversity. This loss is at least partly due to mechanisms operating across spatial scales, such as interference with migration and habitat homogenization, and comes with largely unknown functional consequences. We advocate here for a comprehensive framework for fluvial networks connecting two spatially aware but disparate lines of research on (i) riverine metacommunities and biodiversity, and (ii) the biogeochemistry of rivers and their contribution to the global carbon cycle. We argue for a research agenda focusing on the regional scale—that is, of the entire river network—to enable a deeper mechanistic understanding of naturally arising biodiversity–ecosystem functioning coupling as a major driver of biogeochemically relevant riverine carbon fluxes.

## Introduction

River networks are unique: they are characterized by a hierarchical dendritic structure with unidirectional water flow that imposes constraints on the fluxes of energy, limiting resources, and organisms^1,2^. These **meta-ecosystems** (i.e., collections of local ecosystems connected by flows of material and organisms; detailed definitions of bolded terms can be found in the glossary in Box 1), can only be properly understood in a spatial context. The spatial structure of rivers contributes to their role in the global carbon cycle. Rivers metabolize large amounts of terrigenous OM, producing globally significant carbon fluxes to the atmosphere and ocean^3^. Moreover, river networks can never be treated as a closed system, even as a first approximation. Material and carbon inputs from the terrestrial environment and from groundwater, and outflows to the ocean or the atmosphere are likely to be large relative to flows among locations within the network, especially among those that are not connected by water flow^4^. Thus, understanding terrigenous OM processing in rivers requires considering both the spatial structure of the river network as well as the surrounding **landscape context**.

**Fig. 1:**
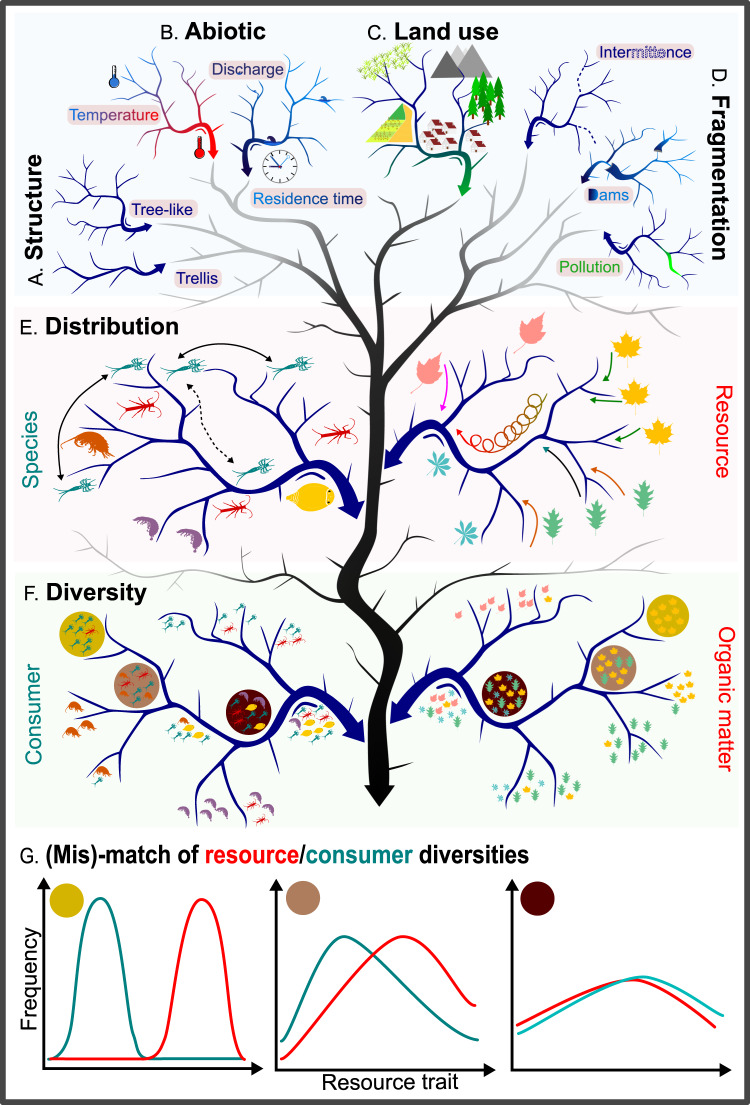
Heterotrophic fluvial meta-ecosystem functioning. **A** The spatial configuration of the river network interacts with **B** the abiotic and physical characteristics of the network, **C** the spatial context in which the network is embedded and, potentially, with **D** topological interruptions to the connectivity of the network. These factors control **E** the distribution and dispersal of consumer species as well as the input and transport of organic matter. Taken together at the network scale, **F** patterns of consumer and resource diversity will emerge. **G** The degree to which these two types of diversity are spatially congruous (and the extent to which human influence disrupts this congruity) will determine meta-ecosystem functioning, and the shape of the BEF relationship.

Respiration in rivers is a heterotrophic **ecosystem function (EF)** that results from consumer **biodiversity** interacting with OM as the main resource. Theory and empirical work have both postulated and demonstrated that biodiversity is a key driver of EF, and that its effects are pervasive and strong^5,6^. However, OM in rivers originates from a range of sources and follows a multitude of transformation pathways while simultaneously being transported downstream^7–9^. For consumers, this results in a chemically complex resource space that changes markedly along a river network^10–12^. The importance of the spatial distribution of consumer biodiversity for the efficiency of OM processing is thus difficult to assess when either consumers or resources are considered alone. Indeed, high **OM diversity** may need considerable (functional) biodiversity of consumers for its efficient processing. Thus, the resource use efficiency for OM, which is a critical measure of EF underlying **biodiversity–ecosystem function (BEF) relationships**^13^, will likely be tied to the capability of locally present organisms to metabolize the locally available OM. Consequently, we expect that while the potential for positive effects of biodiversity on EF exists in most river networks, the degree to which this potential is realized will depend on local conditions, including the abundance and composition of relevant species, competitive equilibria, and the spatio-temporal match between the chemical and physical traits of OM and trophic traits of consumer communities^14–17^. Understanding such river network-wide BEF relationships may be the key to unlocking when and where rivers metabolize OM intensively, thereby supporting fluvial food webs and potentially feeding back to biodiversity, and when inefficient processing rather results in the transport of most OM further downstream (Fig. 1).

OM sources are strongly tied to catchment properties, and OM processing is often viewed through a biogeochemical lens, where transport is a purely physical process and specific knowledge of the local biological community governing OM metabolism is abstracted away or unavailable^18–21^. In contrast, the distribution of organisms in rivers is well-described using metacommunity theory, which explains how dispersal interacts with local conditions to produce species assemblages and aquatic food-webs^22,23^. Indeed, catchment properties can have a strong imprint on the dispersal processes shaping metacommunities, since many organisms disperse with water flow, suggesting that the physical template of the river network acts as a strong underlying control on both community composition and OM distribution. Classically, the river continuum concept^24^ integrates both of these components, describing the dynamics of environmental and biological changes along a longitudinal continuum. It specifically proposes that biological communities are structured by changes in the river’s physical structure and energy sources and availability moving from upstream to downstream, which in turn shapes how these communities use available resources. Yet, in many analyses, a substantial part of the aquatic food web remains unexplained^25^, and the distribution of OM can depend on biological in addition to physical processes^11,26^. Moreover, recent work has shown that a more detailed representation of resource flux in a spatial (i.e., network) context is key for explaining consumer distributions^17^, revealing the limitations of a purely longitudinal view. Importantly, these concepts rarely recognize the importance of the (e.g., chemical) diversity of OM, even though OM represents a critical limiting resource^27–29^.

The spatial configuration of river networks thus constrains the transport of OM and much of the dispersal of organisms, likely leaving a strong imprint on both OM diversity^30,31^ and biodiversity^32–35^ (Fig. 1) because of fundamentally different rules. For instance, organisms can disperse up- and/or downstream depending on dispersal traits, whereas resources are largely transported only downstream. For both, downstream movement depends on both space and water flow; a distant location may be variously more or less accessible depending on flow conditions (e.g., high flow supports fish migration^36^ and accelerates OM transport^20^). Further, the landscape context is key; the composition of the surrounding terrestrial matrix has a large impact on the state of the meta-ecosystem, resulting in multi-scale variation in OM sources, consumer community composition, and the ability of organisms to disperse laterally^37^. Headwaters may be especially sensitive due to their isolation, limited connectivity to other ecosystems and low flow of water relative to more downstream locations^2,38,39^. Downstream regions will further integrate resource and biological inputs from all upstream regions, and so will be sensitive both to the surrounding matrix as well as to disruptions in hydrological connectivity. Anthropogenic influence itself may have a distinct spatial character: for example, point source pollution providing unusually high concentrations of OM and other resources, or fragmentation of the river network by damming and altered flow regimes that may even include non-perennial river network sections.

Here, we advocate for unifying research on carbon biogeochemistry in river networks with that on fluvial metacommunities, focusing on the scale of entire river networks as the primary spatial unit of interest. We briefly review some key research on how both OM biogeochemistry and metacommunities are organized in space, and then argue that future research must consider both simultaneously, especially at the scale of entire river networks, if we are to better understand the coupling between biodiversity and EF and the role of rivers in the global carbon cycle.

## OM as a spatio-temporally dynamic resource space within river networks

**Fig. 2:**
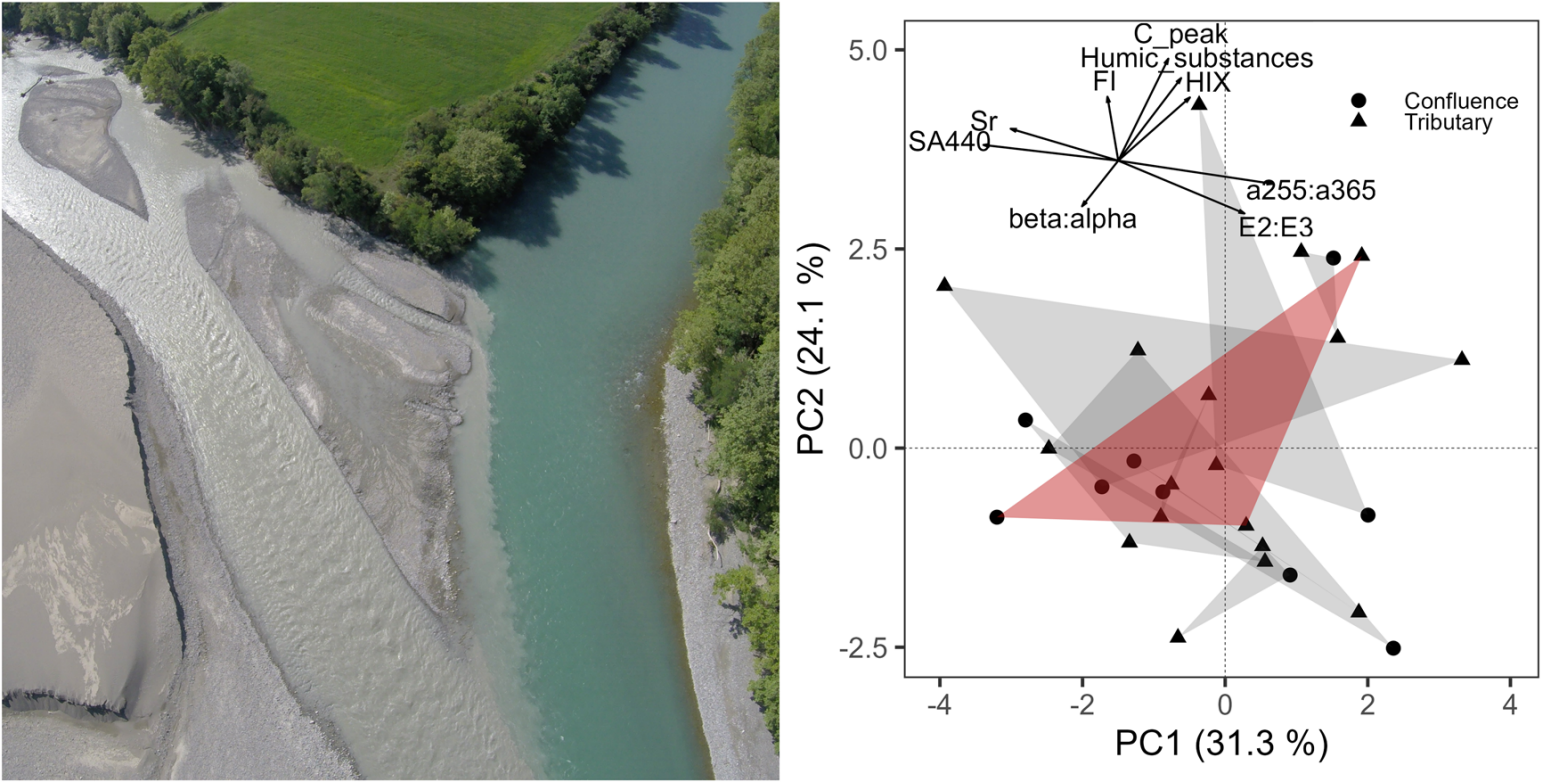
The mixing of river water in confluences draining catchments with contrasting land use or geology can create hotspots of resource diversity. An evident example for this is the confluence of the rivers Sarantaporos (left) and Aoos (right) in the Vjosa river network on the border of Albania and Greece (photo left side, taken in April 2018, credit G. Singer). The huge variation in turbidity reflects upstream differences in geology, and contributes to the diversity in DOM characteristics between the two tributaries. On the right side, a principal component analysis represents the spatial variation in DOM chemistry in the Vjosa river network based on spectroscopic indices. Each polygon represents the differentiation of DOM composition among tributaries and their confluence at various points across the network, with the Aoos–Sarantaporos confluence highlighted in red. The comparison of polygon size gives an idea of the degree of DOM differentiation at every confluence; the differentiation at this confluence of the Aoos and Sarataporos is among the largest across the entire network.

Describing how OM forms a multidimensional resource space for consumers requires advanced capability to describe relevant OM traits. Conceptually, we can divide OM into two pools consisting of dissolved organic matter (DOM) and particulate organic matter (POM), where DOM is <0.45 μm^40^. Indeed, the integration of various OM pools across river networks strongly depends on OM size. DOM moves freely with the water, while for POM particle size controls retention and thus transport behaviour^2^ (Fig. 2). Thus, small particles may have origin points across a larger proportion of the upstream network than large particles (and may also therefore represent OM conditions across a greater portion of the upstream network)^2,41^. Indeed, particle size must be considered as a master trait interacting strongly with other dimensions of the resource space. All environmental, chemical or biological factors involved in OM processing are conditioned by OM particle size^42,43^. Particle size not only mediates how well OM is retained, but also constrains the target consumer community. Since the degradation process of POM also continuously provides new resources (i.e., the formation of smaller detritus fragments), particle size is intrinsically related to the chemical composition and degradability of OM^44^. Thus, the sharp distinction between DOM and POM is somewhat arbitrary; we can more accurately view these two pools as extreme ends of an OM continuum defined by particle size^40^. Here, we consider OM generally as representing the entire continuum, but continue to use the DOM and POM distinction when it is useful for describing different behaviours at opposing ends of this continuum.

Going beyond particle size to understand OM traits requires an understanding of OM composition and diversity. Many studies consider only relatively indirect measures of OM quality. These measures are derived mostly from absorbance or fluorescence spectroscopic analyses of DOM^45^, microscopical identification of fine POM (e.g., relative proportion of animals, diatoms or vascular plant residues)^46^ and selected chemical traits for coarse POM (e.g., C, N, P, lignin, tannins and fibre content)^47^. Novel technology, however, allows us to move beyond such proxies. Indeed, a description of OM diversity should rival the resolution of biodiversity available through molecular biological means and move towards describing actual functional interactions between a consumer and a resource unit (i.e., an organic molecule or particle). For instance, DOM can be described at the level of molecular species by size-exclusion, liquid or ion chromatography coupled to mass spectrometry^48–50^, and POM can be described on a per-particle basis with regard to physical features and macromolecular composition using tools like infrared microspectroscopy^51–53^ (Box 2).

To unravel the role of such highly resolved OM diversity on its processing, we will then need to understand how, where and when OM diversity arises in river networks. DOM molecules may travel for long distances with subsurface soil and groundwater until reaching a headwater stream. DOM begins to degrade along these paths, making it more recalcitrant, but also diversifying the OM pool entering river systems through integration of a larger part of the catchment^54^. In contrast, abscised leaves, likely the most important POM fraction, are sourced locally from riparian vegetation^8,55^. Local sourcing, resulting in a shorter terrestrial path, implies a lower OM diversity, yet a fresher stage when entering the river. Merely given the differences in sourcing pathways from terrestrial systems, DOM and POM diversities at the moment of entering the river network likely depend on the heterogeneity of land cover in the catchment across different spatial scales. Local OM diversity will reflect a combination of these inputs with an integration of similar inputs (and processing) from upstream.

En-route transformation of OM is the final key step to consider in the diversification of the resource space. Discontinuities in the transport of OM can be hot moments (and/or hot spots) of OM processing and therefore, of diversification^3^. Transport discontinuities can happen in space due to fluvial landscape complexity (e.g., in different fluvial habitats of a braided river section or across a floodplain with wetlands), but also in time due to hydrological variability, ranging from the interruption of transport by drying, to fast flushing by flooding. When OM is retained under contrasting environmental conditions, for instance across a transient aquatic-terrestrial habitat mosaic of an intermittent river, it is subjected to different degradation pathways; the result is a diversification of OM without transport, with potential implications for later downstream decomposition when flow and transport are resumed^9,56,57^. The final consequence of these “pulsed” processing-transport dynamics is that OM diversity is not continuously transferred across the river network, but subjected to discrete step changes at confluences.

At large scales this is well conceptualized by the pulse-shunt concept, a transport-dominated view, which is dynamic but ignorant to the level of resource-consumer interaction that we postulate here. In a recent review, Kothawala et al.^58^ point out that OM decomposition requires sufficient water residence time to allow for the interaction of OM and the consumer community^59^, but also the absence of environmental constraints hindering this interaction (e.g., low temperatures or even water availability in drying rivers). We suggest that the lack of a consumer community that is well matched to the resource pool can be as important as the environment for OM decomposition to happen^60^.

## Re-evaluating niche paradigms in fluvial consumer metacommunities: Sorting in the resource space drives consumption

The organization of consumer diversity—that of macroinvertebrates and heterotrophic microbes (bacteria, fungi and protists)—is paramount for structuring OM processing patterns at the regional scale. While river-network scale macroinvertebrate diversity is becoming well documented^32,61–63^, the spatial distribution of microbial diversity is less explored (but see^64–66^). These studies suggest important differences in the mechanisms structuring the consumer metacommunities across river networks. Local-scale consumer diversity is shaped by the interplay of regional processes (e.g., dispersal) and local processes that define the **niche space** and **sort** consumer species from the **regional species pool**^63,67^ (Box 3). The relative importance of regional vs. local processes is highly context dependent, with potential disparities depending the river network structure (including the fragmentation level, dendritic structure, and geology;^17,22,68,69^) and the type of consumer^70^. For example, while microbes generally disperse downstream with river flow, some macroinvertebrates can additionally disperse upstream or overland, depending on their dispersal traits. Notably, metacommunity studies generally suffer from incomplete assessment of the local factors that define niche space^71^, potentially leading to a systematic underestimation of the sorting-driven fraction of community composition. Much research has focused on habitat hydrodynamics, sediment properties, and temperature as key local factors that define niche space, while resources (e.g., OM properties) have remained under-explored. Novel techniques enabling highly resolved characterization of molecular OM diversity and POM size diversity (Box 2) may improve niche space characterization and, ultimately, advance the assessment of the importance of resource diversity in determining consumer community sorting and diversity.

For sorting to be a dominant driver of the consumer metacommunity structure in which OM properties may play a relevant role, a stable environment and low to intermediate levels of dispersal are needed. At high levels of dispersal, regional competition is too strong for local sorting to overcome inputs from dispersal, leading to a community structured by “**mass effects**” (i.e., the incoming mass of upstream migrants overwhelms niche-based selection, and the most common migrants dominate the community)^72–74^. At low to intermediate levels of dispersal the community sorting by local properties is favoured, which can promote greater local consumer specialization, resulting in a mosaic of locally well adapted consumers (Box 3). When confronted with hyper-diverse resources, a poorly adapted consumer community may be simply unable to process the majority of OM, resulting in inefficient OM processing and subsequent OM transport downstream. In contrast, a well-adapted community might be highly efficient at OM processing due to the improved match between resource and consumer traits. Nonetheless, BEF relationships can be saturating^75^; that is, beyond a certain threshold, higher consumer diversity may not lead to higher functionality, as some consumers will have similar functional roles.

Efficient decomposition of chemically diverse OM will only happen where and when there is a proper spatio-temporal match between the features of OM and the traits of local consumers^76,77^. Spatially, the idea of OM “spiralling”^78^ downstream along a flowing watercourse, i.e., the concept of OM of various lability being partly locally consumed and also transformed while simultaneously experiencing downstream transport^79–81^, suggests a pre-programmed mismatch: a community passes on a qualitatively reshaped resource pool that forms the niche space for downstream communities while at the same time sends now less optimally adapted propagules for this consumer community. However, this pre-programmed mismatch may be less relevant for communities that are more sorted by OM derived from local in-stream primary production (i.e., autochthonous OM). In particular for POM, terrigenous inputs can be of low quality, and thus autochthonous POM can be an important resource for invertebrates even when it is not the dominant POM source^82,83^. However, this resource might not be relevant for all invertebrate taxa; shredders (invertebrates that feed by shredding plant material), for example, often exclusively feed on terrigenous POM (see, e.g., ref. ^84^), and are therefore not likely sorted by autochthonous OM.

Temporally, seasonality in OM inputs (i.e., leaf fall) results in changes to the nature of the resource space (i.e., OM properties) over the year. For example, in temperate regions, the majority of allochthonous OM is derived in autumn from the adjacent terrestrial ecosystem because of the high input of POM in form of senescent leaves and the peak of terrestrial DOM washed from the nearby catchment with increasing flows. In contrast, autochthonous OM increases in summer, when temperatures and light availability are highest^85^. Additionally, hydrological variability plays a key role in decoupling resources and consumers. On the one hand, floods enhance terrestrial DOM by washing the surrounding terrestrial ecosystem and mobilizing in-stream retained POM (changing the nature of the resource space) while at the same time scour the communities. In these conditions, most of the OM is transported downstream (i.e., not processed locally), a process known as OM “shunting”^20^. As a result, the OM profile is “flattened” along the river network (i.e, the profile becomes similar across all locations, rather than showing different compositions reflecting variable processing at each location). On the other hand, drying stops the longitudinal flow of resources and consumers, promotes the accumulation of poorly processed OM and impoverishes consumer communities, thereby disconnecting the resource from their potential consumers and enhancing a resource–consumer mismatch. These variations in OM properties interact with the life cycle length/generation time of consumers, which have time scales of days to weeks for microbes but months to years for invertebrates. This timing aligns with the faster variation of DOM, which mostly “goes with the flow”, compared to the “retained” POM properties; but still suggests that consumers always lag behind the dynamics of their resource space. Empirical studies have thus far largely neglected these dynamic resource–consumer interactions^37^ (but see ref. ^17^) and accounting for this asymmetric spatial and temporal variation of OM and consumers will advance the understanding of the dynamics of OM processing at the meta-ecosystem level.

## Research outline and prospectus

We close with a call for research across all scales in fluvial meta-ecosystems, designed to integrate knowledge from the local to the regional scale. First, laboratory experiments will provide the most direct way of manipulating key drivers, such as OM inputs, the available biodiversity, transport and dispersal. Future experiments should go further, both by manipulating aspects of OM and biodiversity in tandem, and by increasing the scale (e.g., by using larger mesocosm facilities, or making manipulations with hyper-diverse communities). Importantly, such experiments are not sufficient to fully characterize regional EF, but they can be used to rule out (or de-emphasize) certain mechanisms in favour of others and can provide estimates about the effect size of various mechanisms.

Second, field studies with adequate regional-scale replication are needed along important natural gradients, especially in understudied regions (e.g., the global south). These gradients can be either spatial (e.g., covering a range of natural network structures, land use gradients, and degrees of human influence), temporal (e.g., pre- and post-damming, time series capturing variable discharge, and intermittence), or ideally both. At even larger scales, we need better efforts to coordinate such studies in multiple river networks to capture continental- and global-scale gradients. This can include the obvious climatic gradients, but should also capture (for example) gradients in human influence and overall degree of landscape heterogeneity, and gradients of fragmentation by damming, water abstraction or flow regimes including drying. Coordinated distributed experiments or global research networks (e.g., eLTER, GLEON) could facilitate the implementation of such large research projects where great sampling effort is unavoidable but unreachable for small research groups or low-funded institutions or countries.

Finally, models will be necessary to bridge the gap between what is possible with laboratory and field studies. In many cases, mechanisms that can be precisely quantified in the laboratory will be infeasible to study in the field, and the degree to which (and mechanisms by which) laboratory-scale effects scale to local ecosystems and regional meta-ecosystems will be unknown or not quantifiable. An added benefit of models is that, when properly constructed, they can themselves provide information back to field studies by generating testable hypotheses and guiding experimental design (Box 4). Ideally, this crosstalk between modelling and empirical work is iterative, where testable hypotheses generated by theory and modelling suggests empirical work, the results of which are used to refine the models.

## Conclusion: Fluvial meta-ecosystems in the Anthropocene

Globally, freshwater ecosystems represent a small fraction of total land area, but support a disproportionately high fraction of total biodiversity and are increasingly under threat^86,87^. We have argued here that threats to biodiversity in rivers represent threats to (meta-) ecosystem function that are potentially much greater than would be predicted under existing frameworks due to the combination of the unique spatial structure of rivers and the potential for biodiversity and resource diversity to become de-coupled in space. These spatial processes include not only the “classic” spatial effects such as land use, network topology, connectivity, and flow, but also the complex spatio-temporal dynamics of organic matter, the biological community, and the spatio-temporal matching of both. It follows that anthropogenic changes will have multi-faceted impacts on fluvial meta-ecosystem functioning, potentially disrupting both consumer and resource stability in the entire meta-ecosystem^88,89^. Therefore, it is essential to understand and integrate mechanisms from local to river network scales. Without this understanding, neither the generation of reliable predictions nor the management for biodiversity and EF in river networks will be possible^1^.

Thus, we call for research that specifically integrates the interactions between consumer communities and OM at all relevant scales in order to properly inform management and assess potential impacts. In particular, it is not enough to consider how a proposed development project will impact local ecosystems, especially when the project will impact connectivity (e.g., diversion projects, dams). Rather, impact assessments must properly consider how work will affect the natural movement of organisms and resources both down- and upstream of the proposed location, and how these regional impacts will propagate to functioning of the entire meta-ecosystem. Moreover, human-induced changes in ecosystem temporal dynamics have the potential to greatly change how consumer–resource interactions unfold in space. For example, changes to resource phenology (e.g., changing timing of leaf fall) will certainly influence the timing of OM availability in the network; such changes will likely propagate to consumer community composition in both space and time and may interfere with consumer phenology^90–92^. Phenological changes could also interact with other temporal anthropogenic changes. Increasing intermittency can increase the accumulation of terrigenous carbon in dry riverbeds, shifting systems away from steady or predictable OM availability and more towards OM pulses^93,94^. The effects of such changes on consumer communities in space and time is understudied. Thus, we argue that only by fully considering spatio-temporal context, feedbacks, and the inter-connected nature of river networks can we close the scale gap and come to a more complete understanding of how and why river networks function the way they do.

## Text boxes

### Box 1. Glossary of bolded terms

**Biodiversity**: The variety of organisms within a specified location. Biodiversity can be with respect to taxonomy (e.g., the number of species), but can also refer to phylogenetic or functional diversity. In the context of this manuscript, biodiversity of feeding traits is particularly relevant for understanding OM processing.

**Biodiversity–ecosystem functioning relationships**: Correlations between the diversity of organisms and the magnitude of ecosystem functions at a location.

**Ecosystem function**: Biophysical processes that contribute to the quantities and fluxes of organisms and materials in an ecosystem. Examples include dispersal rates, primary and secondary production, and nutrient mineralization.

**Landscape context**: A description of the setting in the landscape in which a river network is embedded; for example, the proportion of various land use types, or the geological substrate within a river’s catchment.

**Mass effects**: The influence of species due to large population sizes and/or dispersal fluxes, such that the large number of propagules overcomes other mechanisms structuring a community.

**Metacommunity**: A series of biological communities (i.e., collections of organisms in a particular place in time) that are linked together by dispersal.

**Meta-ecosystem**: Similar to metacommunities; a collection of interconnected ecosystems that exchange material (e.g., nutrients), energy, and organisms via spatial linkages.

**Niche space**: A multidimensional description of the conditions under which a particular organism is able to maintain positive population grown.

**Organic matter (OM) diversity**: The variety of OM in a particular place and time; includes all aspects of OM that might be relevant for consumers, including chemical composition, reactivity, and differences in molecule or particle size.

**Regional species pool**: The collection of species that can potentially occur across an entire metacommunity.

**Sorting**: Community ecology process by which a list of organisms that arrive at a site are selected, generally based on having traits matching the local environmental conditions that allow them to establish and better compete for space and resources.

### Box 2. Measuring relevant niche dimensions: OM diversity defines the resource space for consumers

Chemical composition of OM has been traditionally considered the main driver of decomposition. Most common OM characterization techniques used so far provide only limited biochemical information; either because they only inform about a certain fraction of the OM pool (e.g., chromophoric DOM by spectroscopic measurements), or because the necessity of combining a great variety of laborious chemical analyses to quantify different elements or molecules. This incomplete characterization of OM has resulted in certain knowledge gaps regarding OM processing, such as the lack of understanding of OM molecule interactions controlling priming reactions or non-additive effects of chemical diversity on decomposition^58^. Resolving these knowledge gaps requires that we widen our understanding of OM as forming various niche dimensions. To this end, we must increase the analytical resolution of OM characterization, but also consider additional niche dimensions, e.g., OM physical properties such as particle size).

Novel techniques enable an in-depth chemical molecular characterization of complex OM mixtures, and can be easily applied to OM size gradients. These include nuclear magnetic resonance spectroscopy (NMR)^49^, Fourier transform ion cyclotron mass spectrometry (FT-ICR-MS)^48^ and Fourier transform infrared spectroscopy (FTIR)^50^. However, each of these techniques describes a different chemical structural level of OM. For example, FTIR can only characterize functional groups (e.g., COOH, O–H, C=O); NMR describes chemical bonds (e.g., CH, CH_2_, NH_2_), functional groups and molecules (e.g., carbohydrates, proteins, lipids); and FT-ICR-MS informs about elemental composition (e.g., C, N, O, S) as well as likely macromolecules (e.g., biopolymers, polysaccharides). This includes also chemical bonds, functional groups and simple molecules in OM, but has deficits regarding structural resolution^95^.

Some techniques, such as fluorescence measurements and size exclusion chromatography coupled with organic and/or nitrogen organic detector(s), describe individual molecules and macromolecules and are mostly used to characterize DOM^51^. Although they can also be used in the POM fraction, they require a liquid sample and therefore can only be performed on DOM extracted from the POM, resulting in highly tedious sample processing. On the contrary, pyrolysis gas chromatography mass spectrometry^96^, which describes molecules and macromolecules, is mostly applied to characterize POM. Although it can also be used for DOM, it requires large amounts of freeze-dried sample material.

In the case of POM, techniques such as laser diffractometry, flow cytometry and imaging/photometry are used to additionally characterize the particle physical properties, particularly size. Laser diffractometry is based on the fact that particles, when hit with a light beam, diffract light in a given angle that depends on particle size (i.e., the angle increases with decreasing particle size)^97^. Similarly, flow cytometry detects light scattering after particles pass one by one through incident light beams from one or more lasers^98^. Flow cytometry can be used to measure not only size, but also surface roughness/granularity or volume. Moreover, it can be coupled with fluorescence detectors, allowing automatic differentiation of particle types when some particles are fluorescently stained (e.g., laser in situ scattering and transmissiometry)^99^. Imaging/photometrical techniques, which process particle pictures (obtained, e.g., from microscopes, cameras, etc.) using image processing and particle analysis software (e.g., ImageJ)^100^, can obtain multiple parameters including area (a proxy for size), perimeter, and major and minor axes. While laser diffractometry and flow cytometry are much faster than imaging/photometry techniques when many particles need to be measured, the main advantage of imaging/photometrical techniques is that it can be combined with other techniques (e.g., FTIR) to assess both physical and chemical molecular properties of individual particles.

### Box 3. Metacommunities in river networks: species sorting by environmental factors, dispersal limitation, and chance events

Understanding the distribution, abundance and eventual function of communities is one of the core goals of ecology^67,101^. While much work had been done around well-mixed populations or communities (i.e., single or multi-species assemblages at one site/locality), most natural systems are spatially structured. Spatial structure, and spatial heterogeneity in particular, means that not all species are at all sites all the time, but that there are differences in species distribution and abundance. Presence or absence of species can be due to deterministic factors (e.g., local environmental conditions not allowing a species to survive/persist), species not yet having colonized a site, or chance events (e.g., a species going locally extinct due to a stochastic reason)^102^. In reality, these different processes concur and can generate feedbacks, as the presence or absence of a species can for example trigger the dispersal or survival of another species.

The metacommunity concept describes the processes governing the emergence and persistence of spatially structure multi-species assemblages^67,101^. While environmental factors generally generate the envelope within which each species can persist, colonization of specific locations by species is mostly governed by the interplay of dispersal, persistence, and extinction (due to deterministic or stochastic reasons). Recent work in metacommunity ecology has shown that non-trivial patterns of species distribution and community structure in heterogeneous landscapes can emerge solely due to a combination of dispersal and stochasticity, particularly in river networks^70,103–105^. Importantly, which species is living where in a network of habitats (e.g., along a river network) can be the outcome of the network’s structure and properties, yet also result in feedbacks on community function, for example the complexity or structure of food-webs^17,22^.

Understanding the structure and function of ecological communities requires assessing the abundance and traits of many (potentially thousands) of species across multiple spatial and temporal scales. For example, for a coherent study of realistic food webs that fully mechanistically characterizes the transport and processing of resources, organismal groups including bacteria, invertebrates and vertebrates need to be covered. Until very recently, such monitoring has been largely impossible due to methodological constraints. However, recent advances in the use of environmental DNA (eDNA) techniques allow the study of many organisms at a time by the analysis of their DNA present in water^106,107^, and the reconstruction of communities and food webs across space^108–111^. By doing so, ecology has paralleled (bio)geochemistry with a tool for high-throughput analysis of ecological communities, creating the potential to link organismal composition and diversity to the occurrence and diversity of chemical molecules covering nutrients to complex organic compounds. Ultimately, the integration of these two fields in a spatially explicit perspective may allow understanding of how the abiotic world, shaped by chemical compounds and physical properties, is cross-linked to the biological world, generating feedbacks and ultimately driving the state and properties of both.

### Box 4. Building models of fluvial meta-ecosystem functioning

Models should be an essential component of any research programme studying fluvial meta-ecosystem functioning. In particular, process-based models can be used to explore the intersection between theory and empirical observation and allow for easy scaling from local to regional processes. Models also allow for manipulations that are impractical or impossible in the field, and they can be used as a tool for hypothesis generation, suggesting useful avenues for future field studies. Here, we elaborate a general framework for how such models could be formulated.

We propose that meta-ecosystem models begin from two coupled differential equations, one describing physical state variables, and one biological. As an example, for the physical side, we model the change in the concentration of dissolved organic carbon ($\left[{\rm {DOM}}\right]$) in a stream reach *i* using a transport-reaction equation^112^:

1 $$\frac{\partial {\left[{\rm {DOM}}\right]}_{i}}{\partial t}={\rm {input-output}}-\mathop{\sum }\limits_{j=1}^{s}{\rm {consumptio{n}}}_{j}\times {\rm {abundanc{e}}}_{j}$$

where input is the combination of transport of DOM from upstream reaches and contributions from lateral (e.g., overland or groundwater) inputs, and output is DOM transported to the next reach downstream. These terms will necessarily scale with discharge, and may also be modelled as a function of other characteristics (e.g., streambed area, leaf production) depending on the level of detail required. The final term is reaction, summed across all *s* species present in the reach, and combines the consumption rate of a species *j* and the abundance of that species in the reach. This equation operates at the local scale; regional dynamics emerge from the interactions between transport (driven by connectivity and water flow in the river network) and the consumption of resources, which is itself determined by metacommunity dynamics.

For the community side, we can describe the rate of change in the number of reaches occupied by species *j*:

2 $$\frac{\partial {P}_{j}}{\partial t}={P}_{j}\left(N-{P}_{j}\right){c}_{j}\left(\left[{\rm {DOM}}\right]\right)-{P}_{j}{m}_{j}\left(\left[{\rm {DOM}}\right]\right)$$

This is a simple metapopulation model^113^, but extended to include all species in a community. The first term in the model describes colonizations; *P*_*j*_ is the number of reaches occupied by species *j* and serves as a dispersal term (i.e., more occupied reaches results in more colonization). *N* is the total number of reaches; we multiply by (*N*−*P*_*j*_) because sites must be unoccupied to be colonized. Finally, *c* is the colonization rate, which is a function of the DOM concentration, thereby linking it to the physical equation. The second term describes extinctions and follows similar logic; a reach must be occupied to experience extinction, and the extinction rate *m* is a function of the DOM concentration.

Importantly, colonization and extinction rates are heterogeneous in space, shifting the focus of the model from the regional scale to the local scale. Other processes of interest can also be easily incorporated; for example, competition with other species can be incorporated in the extinction term^114^, dispersal rates can influence colonization^115^, and habitat/niche dimensions can be made a part of the colonization and extinction functions^116,117^.

These two models are strongly linked; the processing term in the resource equation is dependent on the traits of locally available species and will be higher when species possess the ability to process the resource. Conversely, species with high affinity for the resource will be more likely to colonize a reach where the resource concentration is high, and more likely to go extinct when it is low. Meta-ecosystem functioning is an emergent property in this model that results from the interplay of species presence–absence across all reaches and the DOM consumption rates of these species. It can easily be estimated by summing the resource processing term over all local ecosystems and over a desired time interval.
